# Natural Matrine‐Integrated Pollen Delivery Systems for Allergic Contact Dermatitis Treatment

**DOI:** 10.1002/smmd.136

**Published:** 2025-02-26

**Authors:** Yuwei Wang, Lijun Cai, Yuanyuan Zhang, Yan Cong, Yuanjin Zhao

**Affiliations:** ^1^ Department of Rheumatology and Immunology Nanjing Drum Tower Hospital Clinical Medical College of Traditional Chinese and Western Medicine Nanjing University of Chinese Medicine Nanjing China; ^2^ Department of Rheumatology and Immunology Lianyungang Affiliated Hospital of Nanjing University of Chinese Medicine Lianyungang China; ^3^ School of Biological Science and Medical Engineering Southeast University Nanjing China; ^4^ Department of Dermatology and Venereology Nanjing Drum Tower Hospital Affiliated Hospital of Medical School Nanjing University Nanjing China

**Keywords:** allergic contact dermatitis, Chinese medicine, drug delivery, matrine, pollen, skin disease

## Abstract

Allergic contact dermatitis (ACD) is an inflammatory dermatitis with a high morbidity and recurrence rate. Scientific attention is focused on the development of safe and comfortable therapeutics of ACD. Herein, we propose a natural matrine‐integrated pollen delivery system for the ACD treatment. Sunflower pollens were collected and defatted to serve as adhesive drug carriers for matrine. Specifically, the exquisite porous and hollow structures of the pollen shells can absorb matrine and realize the sustained drug release. Besides, the prickly surface morphology can strongly adhere to the inflamed skin sites, which can prolong the duration of the drug. By utilizing them in an ACD model and an acute pruritus model of mice, we have demonstrated that these matrine‐integrated pollen shells can decrease the swelling degree of mice ears and weight loss, down‐regulate inflammatory response, and improve the scratching times. These results indicate that our matrine‐integrated pollen delivery systems have great potential to serve as natural topical preparations for skin disease therapy.

## Graphical abstract

Novel matrine‐integrated pollen delivery systems were utilized to treat allergic contact dermatitis (ACD). The prickly porous structure of matrine‐integrated pollen shells could adhere to the lesion site to sustained release drugs, prolonging the duration time of matrine, indicating the great potential to serve as natural topical preparations for ACD therapy.

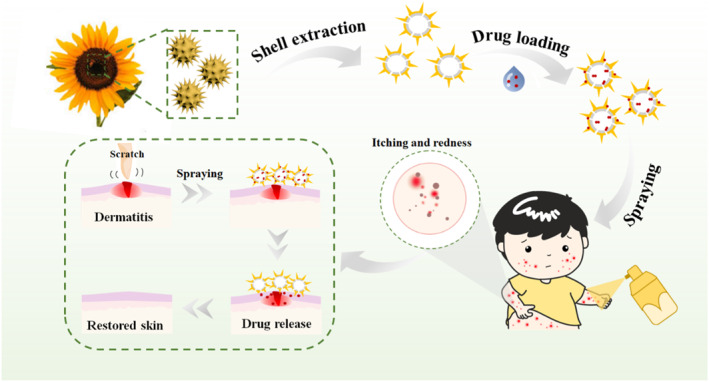

**Summary**

- The microspheres containing spinous porous structures are successfully fabricated from sunflower pollen grains, which have excellent adhesion and sustained release ability.
- Matrine‐integrated pollen delivery systems are developed for allergic contact dermatitis (ACD) therapy.
- The matrine‐integrated pollen delivery systems inhibit acute pruritus and reduce inflammation in the ACD mice model.

## 1 Introduction

Allergic contact dermatitis (ACD) is a common skin disease of eczematous dermatitis, whose pathogenesis includes impaired skin barrier function, immune disorder, inflammatory response, and so on [1, 2, 3, 4, 5]. As the symptoms of ACD involving local redness, rash and itching cause lasting suffering to patients, various therapeutic methods have been proposed for the treatment of ACD [6, 7, 8, 9, 10, 11, 12]. Among them, applying an ointment to the lesion area is the most widely used way [13, 14, 15]. These ointments containing low to moderate potency corticosteroids and glucocorticoids have been proven effective in treating the ACD [16, 17]. However, recurrent seizures of ACD and the long‐term medication may have side effects such as infection and skin atrophy [18, 19]. In addition, most ointments are added with synthetic fragrances and preservatives, which may lead to skin sensitization, thus deteriorating ACD [20, 21]. Therefore, conceptions on how to develop drug delivery systems that can prolong the duration of the drugs while reducing side effects will be of far‐reaching significance on the development of treating ACD [22].

**FIGURE 1:**
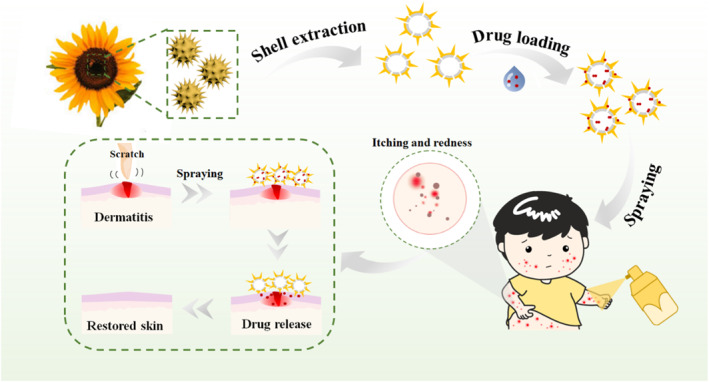
Schematic illustration of natural matrine‐integrated pollen delivery systems for allergic contact dermatitis treatment.

In this paper, we present a novel natural matrine‐integrated pollen delivery system for dermal administration for efficient treatment of inflammatory dermatitis, as schemed in Figure 1. Matrine is the main material basis of Radix Sophora Flavescentis [23]. As a typical kind of natural Chinese medicine, Radix Sophora Flavescentis has been proven to have strike effects of anti‐inflammation, anti‐bacteria and regulating immunity, exhibiting good therapeutic effects on inflammatory skin diseases such as ACD [24, 25, 26, 27, 28]. Inspired by the properties of nature, the mining and utilization of natural biomaterials have an important impact on the development and innovation of pharmaceutical dosage forms. Compared with synthetic organic materials, the treated pollen shells (TPSs) are strong and elastic, which can reduce the oxidative denaturation of the drug through the cavity to improve the bioavailability of the drug. In contrast, natural pollen grains are characterized by a hollow cavity, robust shell and strong adhesion, serving as promising carriers for drug delivery [29, 30, 31, 32, 33, 34, 35]. Therefore, it's believed that an effective treatment method for ACD can be devised via the integration of matrine into pollen shells.

In this paper, we loaded the matrine into the pollen shells for the ACD treatment by chemical defatting and physical absorption. The pollen shells were collected from natural sunflowers, and their cement and sensitizing components were removed using chemical methods to expose the porous surface and shed the immunogenicity. Due to the exposed porous surface and inherent hollow structure, these pollen shells could be effectively loaded with matrine via physical absorption. Besides, benefiting from their unique spiny architecture, the matrine‐integrated pollen shells (MPSs) could adhere to the surrounding skin surface, which would prolong the duration and thus enhance the bioavailability of matrine. To demonstrate their practical value, we evaluated the therapeutic effect of MPSs on the acute pruritus model and ACD model of mice. It's proved that the MPSs could efficiently relieve the symptoms of ACD including edema, crusting, weight loss and pruritus profiting from their outstanding properties of adhesion and drug delivery, unveiling broad prospects in skin disease treatment.

## 2 Results and Discussion

**FIGURE 2:**
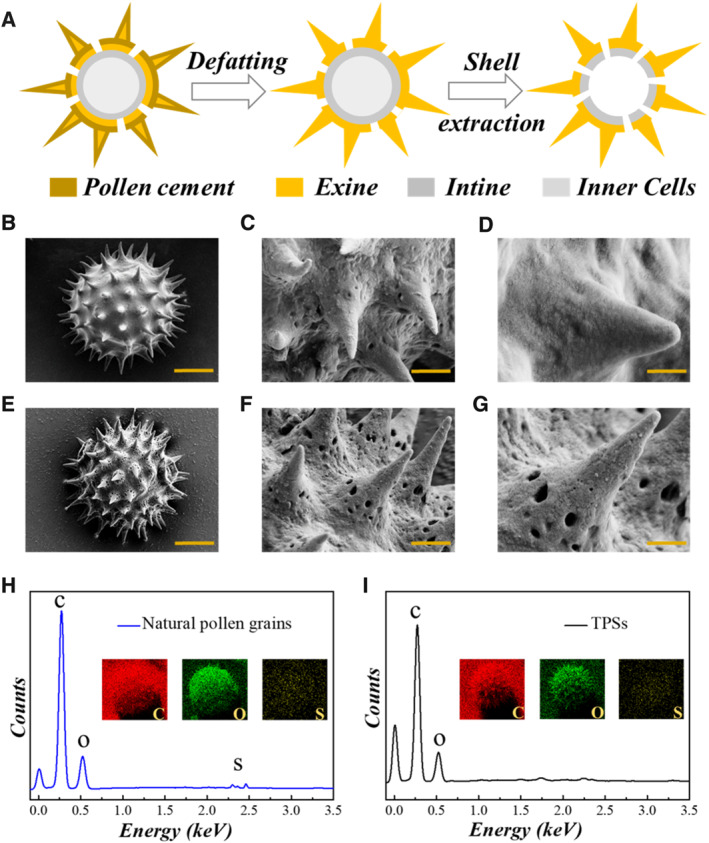
Preparation and characterization of TPSs. (A) The preparation process of TPSs. (B–D) SEM images of natural pollen grains. Scale bars are 10 μm in (B), 2 μm in (C) and 1 μm in (D). (E–G) SEM images of TPSs. Scale bars are 10 μm in (E), 2 μm in (F) and 1 μm in (G). (H, I) EDS spectra of natural pollen grains and TPSs. The inner figures are EDS mapping of natural pollen grains and TPSs.

In the typical experiments, the sunflower pollen grains were utilized to prepare drug carriers (Figure S1A–C). The surface morphology of pollen grains was analyzed by a scanning electron microscope (SEM). It was observed that there were natural microprotrusion structures on the outer layer of pollen grains, while the porous texture was covered by the cement of natural pollen grains (Figure 2B–D). Then, the cement of natural pollen grains were removed to expose the porous structure via defatting and chemical hydrolysis (Figure 2A). The natural pollen grains were dispersed with ultrapure water, degreased with organic solvent, and hydrolyzed with strong alkaline to eliminate the internal contents. From the SEM images of treated pollen shells (TPSs), the spiny structure was preserved after defatting and chemical hydrolysis, while the porous structure was fully displayed, indicating that the pollen shells with spiny porous hollow structure were obtained (Figure 2E–G). The unique structure of the pollen shells endowed them with large surface‐to‐volume ratio, making them ideal as drug carriers. Through Energy Dispersive Spectrometer (EDS) mapping images in Figure 2H,I, elements including C, O and S could be detected in the natural pollen grains, while only two basic elements (C and O) were detected after defatting and chemical hydrolysis, which indicated that protein substances that were pollen allergens were successfully removed, making them safe as drug carriers. In summary, TPSs with spiny hollow structures were obtained and served as promising carriers for drug delivery.

Due to the unique spiky structure, the TPSs were expected to possess adherence property. To verify this, the retention rate of TPSs on tissues was compared with that of spherical microspheres of the same size. To be specific, equal numbers of pollen shells and spherical microspheres were exposed to the skin surface and washed by flows. As depicted in Figure S2A,B, after flow rushing, more pollen shells attached to the skin compared with spherical microspheres. Furthermore, the retention rate of pollen shells was twice as high as that of spherical particles (Figure S2C). These indicated that the pollen shells had excellent adhesion ability benefiting from their unique spiny structure, making them promise for durable drug delivery.

In this experiment, the TPSs were loaded with matrine, which is the main material basis of Chinese medicine Radix Sophora Flavescentis, for treating ACD by simply immersing them in the solution of matrine. To explore the loading and releasing capacity of the resultant pollen shells, rhodamine B (RhB) was chosen as a drug model due to its notable detectable signal. RhB was loaded to the TPSs by soaking in the aqueous solution of RhB. As shown in Figure S3A, the higher concentration of RhB contributed to the higher loading efficiency. Furthermore, vacuum treatment was utilized during the loading process. It's found that vacuum treated group exhibited higher loading efficiency than the passive loading group. Meanwhile, a loading duration of 15 min could achieve the maximum loading efficiency (Figure S3B). Thus, vacuum treatment for 15 min was utilized to load the drugs in the following experiment. For further testing and verifying the drug release ability of TPSs in vitro, the RhB‐loaded TPSs were exposed to phosphate buffered saline (PBS). The fluorescence intensity of RhB‐loaded TPSs decreased over time (Figure S4A), indicating the sustained release of RhB from TPSs. The cumulative release curve of RhB was recorded in Figure S4B, which revealed that the RhB‐loaded TPSs released the RhB quickly within one day. After 12 h, the cumulative release ratio showed a slow upward trend, and the maximum cumulative release rate was about 66% after 48 h. All those results proved that our TPSs could be used as ideal vehicles for drug delivery based on their excellent drug loading and releasing capacity.

**FIGURE 3:**
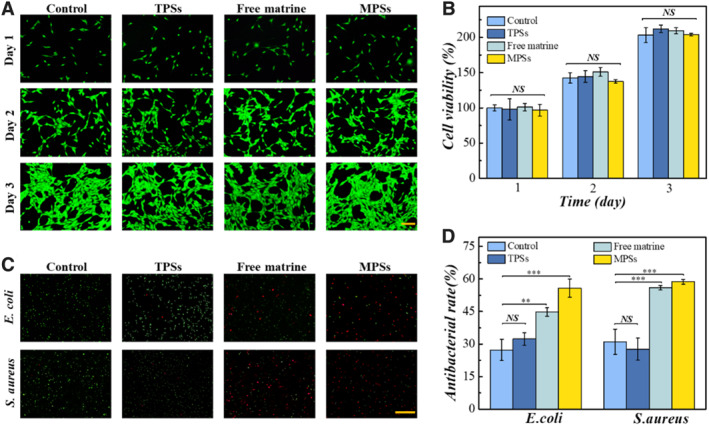
Cytotoxicity and antibacterial properties of MPSs. (A) Live staining images of NIH 3T3 cells. (B) Cell viability of NIH 3T3 cells. (C) Images of live and death staining of *Escherichia coli* and *Staphylococcus aureus*. (D) Antibacterial rate of different groups. Scale bars are 100 μm in (A) and 50 μm in (C). **P < 0.01; ***p < 0.001; NS, not significant.

Considering safety is a fundamental property of drug carriers, biocompatibility test and hemolysis test were carried out for evaluating the biosafety of the TPSs [36]. Firstly, the cell cytocompatibility of TPSs, free‐matrine and matrine‐integrated pollen shells (MPSs) were tested by co‐culturing them with NIH 3T3 cells. As depicted in Figure 3A, the cell morphology in all experimental groups was similar to the control group. Furthermore, the survival of NIH 3T3 cells in each group was determined by MTT assay. As manifested in Figure 3B, the cell viability of all groups reached about 200% on day 3. These results showed that the NIH 3T3 cells in all groups still maintained good biological morphology and growth, exhibiting satisfactory biocompatibility of MPSs. Subsequently, the blood compatibility of TPSs was verified by hemolysis test. The hemolysis rate was determined by co‐culturing the MPSs with rat erythrocytes. Through the optical images in Figure S5A, the MPSs did not cause obvious hemolysis. Moreover, the hemolysis rate of MPSs was 2%, which demonstrated excellent blood compatibility (Figure S5B). Generally, the MPSs have great potential in the treatment of inflammatory skin diseases benefiting from their excellent biological safety.

After that, we further investigated the antibacterial capacity and the antipruritic capacity of MPSs, which could resist bacterial infections and relieve the acute itching aroused by ACD, thus promoting the restoration of normal physiological state and function of skin tissue. The sterilization properties of MPSs were investigated with *Escherichia coli* (*E. coli*) and *Staphylococcus aureus* (*S. aureus*), which were used as model bacteria. To be specific, the bacteria were divided into four groups, which co‐cultured with PBS, TPSs, free matrine, and MPSs for 24 h, respectively. Then, the bacteria in each group were conducted dead and live staining, and fluorescence images were taken to observe the bactericidal effect. As shown in Figure 3C,D, the bacteria in the MPSs were decimated, and the antibacterial rate reached about 68%. These results indicated that these MPSs could inhibit skin ulcers and bacterial growth, which could promote the recovery of ACD.

To evaluate the antipruritic ability of MPSs, a mice acute pruritus model was established by a combination of histamine and 4‐Aminopyridine (4‐AP). The latent time and scratch times of the mice are recorded in Figure S6A,B. It's found that the latent time was significantly prolonged in the free matrine and MPSs treated groups compared with the control group. Besides, the times of itching occurrence were significantly reduced in the free matrine and MPSs treated groups. Moreover, the improvement of the MPSs treated group was more obvious than that of the free matrine treatment group. These data demonstrated that the MPSs could significantly improve the therapeutic outcomes profiting from the longer retention time, which could benefit the recovery of ACD.

**FIGURE 4:**
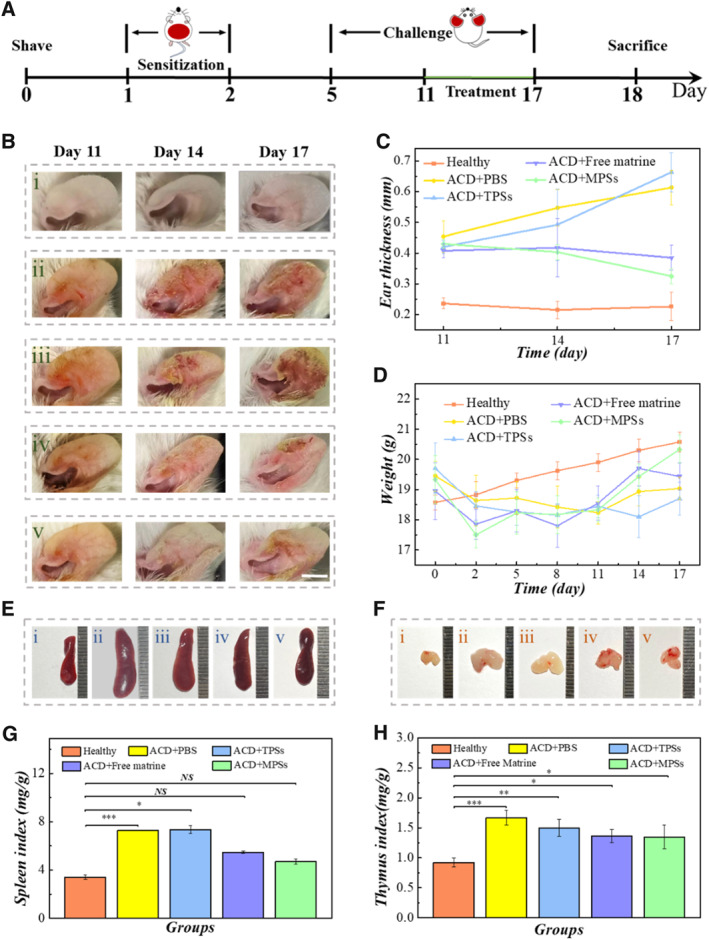
Therapeutic effect of MPSs on ACD. (A) Experimental flow chart of ACD mice model establishment and treatment. (B) The appearance of mice ears in different groups. (C) Statistic analysis of the ear thickness. (D) Weight change of mice during the experiment. (E) Images of mice spleens. (F) Images of mice thymuses. (G, H) Statistical analysis of spleen index and thymus index from different groups. The groups in (B), (E) and (F) are healthy mice (I), ACD mice treated with PBS (ii), TPSs (iii), free matrine (iv), and MPSs (v). Scale bar is 5 mm in (B). *P < 0.05; **P < 0.01; ***P < 0.001; NS, not significant.

To further evaluate the practical application ability of the MPSs in vivo, we used them to treat mice with an ACD model. First, the mice were divided into five groups, including healthy control group and four diseased groups, which were treated with PBS, TPSs, free matrine and MPSs, respectively. As shown in Figure 4A, all diseased mice were induced to ACD by repeated application of 2,4‐dinitrofluorobenzene (DNFB) to the bare skin. To be specific, the DNFB diluent was smeared on abdominal skin and bound to the skin protein to form a complete antigen, stimulating the proliferation of T lymphocytes into sensitized lymphocytes. Then, the DNFB solution was applied to the ears again, resulting in swelling and scab. After successful modeling of ACD, the mice were given with the treatment of PBS, TPSs, free matrine and MPSs from day 11 to day 17 and challenged with DNFB at the ears every 3 days, respectively. The changes in ear thickness and weight in the modeling and administration process were recorded. As shown in Figure 4B,C, with every challenge, there was no significant thickening and deterioration of the ear in the diseased group of MPS treatment. In comparison, the thickness of the ear increased obviously in the other diseased groups. Meanwhile, the thickness of the diseased group treated with PBS and TPSs increased more than those treated with free matrine.

Moreover, the weight of the four diseased groups showed an obvious decreasing trend during the modeling period, while that of the healthy control group increased steadily (Figure 4D). Besides, the weight of the diseased group treated with PBS and TPSs kept a decreased trend with administration. Conversely, the diseased group treated with free matrine and MPSs gained weight. Exactly, the diseased group treated with MPSs had a greater increase in weight compared to that treated with free matrine.

Aiming at reflecting the therapeutic effect intuitively, the spleen and thymus of all mice were collected on day 18. As shown in Figure 4E–H, the spleen index and thymus index of diseased mice that received treatment with PBS were much higher than that of the control group, which may be due to the immune response induced by DNFB, thus leading to the enlargement of immune organs. However, the organ index decreased in diseased groups that treated with free matrine and MPSs. Notably, the organ index of diseased mice with MPSs treatment presented a pronounced improvement, which was more closely matched with control group, indicating a superior efficacy than free matrine.

**FIGURE 5:**
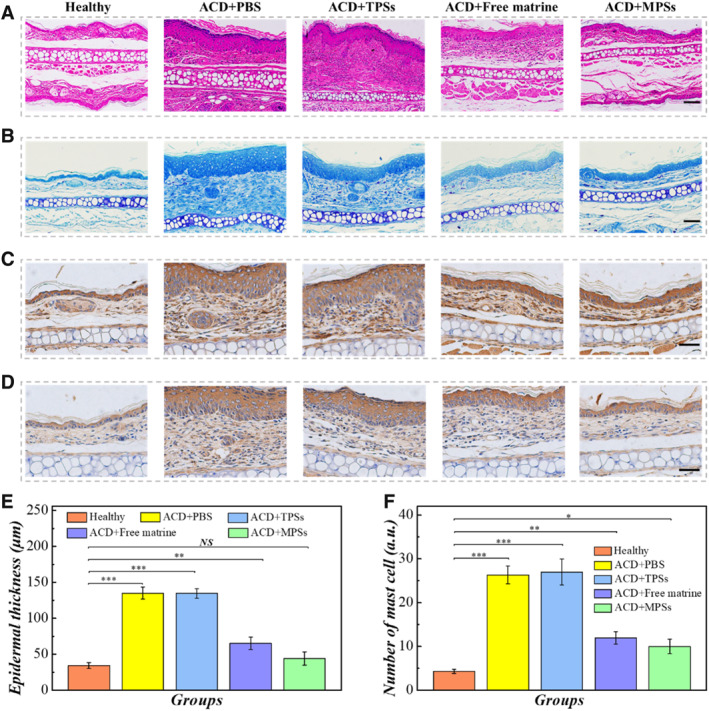
Healing effect and immunohistochemical staining of mouse ears in different groups. (A) H&E staining images of mouse ears in different groups. (B) Toluidine blue staining images of mice ears in different groups. (C) IL‐1β staining images of mice ears in different groups. (D) TNF‐α staining images of mice ears in different groups. (E) Statistical analysis of epidermal thickness. (F) Statistical analysis of mast cell infiltration. Scale bars are 50 μm in (A) and (B), 40 μm in (C) and (D). *P < 0.05; **P < 0.01; ***P < 0.001; NS, not significant.

Moreover, we carried out histopathological analysis of these collected ears for further evaluation of the therapeutic efficacy. Hematoxylin and eosin (H&E) images of mouse ears in five groups on day 18 are shown in Figure 5A,E. There were severe keratosis and numerous inflammatory cells in the ear of diseased mice treated with PBS and TPSs, and the epidermal thickness increased compared with the healthy group. After the medication, the inflammatory cell infiltration was significantly inhibited in the diseased group treated with MPSs compared with the free matrine treatment group. Meanwhile, the epidermal thickness of the ear showed a larger diminution with the MPS treatment than that with free matrine treatment. Mast cells were identified by toluidine blue (TB) staining (Figure 5B,F); a serious edema degree and mast cell accumulation were observed in the diseased group with PBS and TPS treatment. Treatments with free matrine and MPSs resulted in a reduction in mast cell infiltration. Precisely, the MPSs group showed similar results with the control group, further suggesting that MPSs were more effective in therapy.

Immunohistochemical methods were used to detect the expression of interleukin‐1β (IL‐1β) and tumor necrosis factor‐α (TNF‐α) in the ear tissue, which could directly mediate the ACD. As shown in Figure 5C,D, it was found that expression levels of IL‐1β and TNF‐α were increased in the diseased group that received PBS and TPS treatment. However, the MPSs could inhibit the expression of these cytokines (Figure S7A,B) and had better restorative effect of ACD than free matrine profiting from their excellent property of drug delivery and adherence, which showed great potential for clinical applications.

## 3 Conclusion

In general, we developed a natural matrine‐integrated pollen delivery system to improve the bioavailability and reduce the side effects of drugs by encapsulating the matrine into pollen shells. Firstly, the natural pollen grains were defatted to expose the unique spiny, porous and hollow architecture, forming adhesive carriers with capacity of sustained release. Compared to the free matrine group, these matrine‐integrated pollen shells showed satisfactory biocompatibility and superior bactericidal properties in vitro. To further explore their practical value in vivo, we established the acute pruritus and ACD models in mice. It has been proven that the latency time for itching in mice was effectively prolonged while the itching frequency was notably reduced. Additionally, the ear epidermal thickness, as well as the number of mast cells and inflammatory factors, decreased in the ears of ACD mice. These results all indicated that the matrine‐integrated pollen delivery system possessed superior antipruritic and anti‐inflammatory properties compared to the free matrine group, which was associated with the prolonged duration of action and higher drug bioavailability. These results fully demonstrated the safety and effectiveness of the designed drug delivery system, which could be a favorable candidate for the clinical skin disease therapy.

## 4 Experimental Section

### 4.1 Materials

Matrine, 2,4‐dinitrofluorobenzene (DNFB), histamine and 4‐Aminopyridine (4‐AP) were obtained from Shanghai Aladdin. Rhodamine B (RhB) was supplied from BASF Chemical Co., Ltd. Servicebio Co., Ltd. supplied phosphate buffered saline (PBS), saline and paraformaldehyde. The methyl thiazolyl tetrazolium (MTT) kit and Luria‐Bertani (LB) broth medium were brought from ThermoFisher Scientific. Calcein‐AM was supplied from Molecular Probes Co., Ltd. SYTO, propidium iodide (PI) and dimethyl sulfoxide (DMSO) were obtained from Macklin. Olive oil was bought from Zhongliang Food Marketing Co., Ltd. The Cell Bank of the Chinese Academy of Sciences (China) provided NIH 3T3 cells. Besides, the BALB/C mice were obtained from Drum Tower Hospital, and the Animal Investigation Ethics Committee of Nanjing Drum Tower Hospital permitted the animal experimental programs (2024AE01054).

### 4.2 Fabrication of Pollen Shells

Natural pollen grains (250 g) and acetone (500 mL) were mixed and refluxed at 50°C for 3 h with stirring at 220 rpm. Then, the mixture was separated by vacuum filtration. The collected sample was then treated with acetone as described above, and filtrated. Next, 20 g sample was soaked in diethyl ether (250 mL) for 2 h and stirred at 400 rpm. The diethyl ether treatment step was performed three times, and fresh diethyl ether was added after each vacuum filtration. Among them, the mixture was stirred overnight in the final diethyl ether treatment step. After that, the pollen mixture was filtered and dried in a fume hood. Next, 2 g of the resultant sample was immersed in 10% (w/v) KOH (20 mL) at 80°C for 2 h and stirred at 800 rpm. Then, the sample was washed by fresh KOH (40 mL) for 5 times to obtain the TPSs.

### 4.3 Test of Adhesion in Vitro

RhB‐loaded TPSs were chosen as the experimental group to test the adhesion performance of pollen shells in vitro. Meanwhile, the black ink‐loaded spherical particles, which have the same size as RhB‐loaded TPSs, were used as the control group. Then, the suspensions which had the same mass of the spherical particles and RhB‐loaded TPSs (Ninitial) were placed on the mice skin and flushed with PBS for 2 and 4 times to remove the unattached particles. The number of particles adhered to the skin was recorded (Nstayed). The following equation was developed to calculate the retention rate:

(1) $$\text{Retention}\;\text{rate}\;\left(\%\right)=\frac{N_{\text{stay}}}{N_{\text{initial}}}\times 100\%$$

### 4.4 Test of Loading Efficiency

RhB was selected as the model of the small molecule drug. The same number of TPSs was soaked in different concentrations of RhB solutions (1, 5 and 25 mg/mL) for 15 min. Meantime, the TPSs were mixed with RhB solution (25 mg/mL) and kept in a normal or vacuum environment for 1, 5 and 15 min. After washing and lyophilizing all the RhB‐loaded TPGs, an equal number of RhB‐loaded TPSs were ground, suspended with PBS, and centrifuged to collect the supernatant. Then, the supernatant was placed in a 96‐well plate and scanned at 570 nm, defining unloaded TPSs as blank groups. The amount of RhB loaded in the RhB‐loaded TPSs could be calculated. The loading efficiency of RhB in the different groups were calculated using the following equation:

(2) $$\text{RhB}–\text{loading}\;\text{efficiency}\;\left(\%\right)=\frac{\text{RhB}\;\left(\text{mg}\right)}{\text{RhB}-\text{loaded}\;\text{poleln}\;\text{shells}\;\left(\text{mg}\right)\;}\times 100\%$$

### 4.5 Test of Drug Delivery

The pollen shells were soaked in RhB solution and placed in a vacuum environment for 15 min to obtain the RhB‐loaded TPSs. After washing and remixing with PBS, the RhB‐loaded TPSs were incubated at 37°C. Then, 100 μL of the upper liquid was collected periodically in a 96‐well plate. Meantime, an equal amount of fresh PBS was added. Then, scanning the 96‐well plate at 570 nm.

### 4.6 Test of Biocompatibility

NIH 3T3 cells were inoculated and cultured in a 24‐well plate (105 cells in each well). PBS, TPSs, free matrine and MPSs were co‐cultured with culture medium for 24 h in advance. After filtration, the co‐culture medium was added to wells with NIH 3T3 cells and incubated for 1, 2 and 3 days. The wells that added PBS were considered as the control group. MTT was utilized to each well and then incubated for 4 h. After removing the MTT, 800 μL DMSO was added in each well, and the absorbance was determined at 490 nm. Calcein‐AM was utilized to stain live cells and then a fluorescence microscope was utilized to observe the cells.

### 4.7 Hemolysis Test of MPSs

Rat blood was collected. Then, the samples were centrifuged at 1500 rpm for 10 min. The centrifugation step was repeated three times. With every centrifugation, the supernatant was discarded, and saline was added. Then, the collected red blood cells were tailored into 2% (v/v) red blood suspension with saline. Saline, ultrapure water and MPSs were added to the red blood suspension (2 mL) and set overnight at 4°C. Then, the mixture was centrifuged again to collect the supernatants to scan at 570 nm. The calculation was based on the formula in Figure S5B.

### 4.8 Test of Antibacterial in Vitro

*E. coli* and *S. aureus* were proliferated and suspended in LB broth medium. To adjust the concentration to MacFarlane's standard turbidity (0.5), the bacterial suspension was diluted with PBS. Then, PBS, TPSs, free matrine and MPSs were added into wells and incubated with 500 μL bacterial liquid, respectively. After 12 h, SYTO and propidium iodide (PI) were added to stain the live/dead bacteria. Images were recorded using an upright fluorescence microscope.

### 4.9 Establishment of Pruritus Model of Mice

A BALB/C mice pruritus model was established by a combination of histamine and 4‐AP. On day 0, the hair on the mice neck skin was shaved. On days 1–7, 50 µL of PBS, TPS suspension, free matrine (1.2 mg/mL) and MPS suspension were individually painted on the shaved neck. 7 days later, 50 μL of mixed solution of 0.05% (v/v) histamine and 0.02% (w/v) 4‐AP was injected into the neck. The behavior of mice was observed immediately. To evaluate the therapeutic efficacy on pruritus, the latent time of itching and scratch frequency were observed immediately after 15 min.

### 4.10 Establishment of ACD Model of Mice

ACD was induced through the repeating application of DNFB. BALB/C mice were divided into one healthy control group and four ACD model groups. On days 1 and 2, 20 µL of acetone/olive oil (4:1) solution containing 7% (v/v) DNFB was applied to the shaved abdomen of mice in the model group for sensitization. On days 5, 8 and 11, the mice were painted 20 μL 1% (v/v) DNFB solution on the ears for challenging. On day 11, the ears of mice showed swelling and scab, and the ACD model of mice was successfully established.

### 4.11 Evaluation of the Therapeutic Efficacy of MPSs on ACD

After successful modeling, the drug was administered continuously for 7 days. The mice in the control group were fed normally without intervention. The other groups were treated with 50 μL of PBS, TPS suspension, free matrine (1.2 mg/mL) and MPS suspension on ears. On days 14 and 17, the 20 μL of 1% (v/v) DNFB solution was repainted on the ears and the ear thickness of mice was measured 5 h after each challenge. On day 18, the mice were sacrificed to excise the ears, spleens and thymuses, which were then fixed for further tissue staining and analysis. The ear thickness and weight of the mice were recorded during modeling and treatment process.

### 4.12 Histopathology

Four percent (v/v) paraformaldehyde was used to fix the ears of mice and then the paraffin‐embedded ears were sliced. H&E staining was utilized to analyze the slices and the thickness of the epidermis was measured. TB staining was utilized to measure the mast cell infiltration. Immunohistochemical staining was used to observe the infiltration of inflammatory cells, and the area of positive coverage was counted.

### 4.13 Characterization

An optical microscope (OLYMPUS BX51) was utilized to take optical photographs of pollen grains and stained sample sections. The characterization of pollen microstructure was carried out by scanning electron microscope (SEM, Hitachi S‐3000N). Fluorescence photographs were recorded using a fluorescence microscope (OLYMPUS IX71). ImageJ was utilized to quantitatively analyze photos.

## Author Contributions

**Yuanjin Zhao:** conceptualization, funding acquisition, supervision. **Yuwei Wang:** investigation, methodology, writing–original draft. **Lijun Cai, Yuanyuan Zhang** and **Yan Cong** assisted with manuscript writing–review and editing.

## Ethics Statement

The animal experiments have received approval from the Animal Investigation Ethics Committee of Drum Tower Hospital (2024AE01054).

## Conflicts of Interest

The authors declare no conflicts of interest.

## Supporting information

Supporting Information S1

## Data Availability

The authors have nothing to report.
