# Supplementary material for: Natural Matrine‐Integrated Pollen Delivery Systems for Allergic Contact Dermatitis Treatment
Source: Smart Med. 2025 Feb 26;4(1):e136. doi: 10.1002/smmd.136 (PMC11862104; doi:10.1002/smmd.136)
Supplement: Supplementary file 1 — Supporting Information S1 [file SMMD-4-e136-s001.docx]

Supporting Information

Natural matrine-integrated pollen delivery systems for allergic contact dermatitis treatment

Yuwei Wang, Lijun Cai, Yuanyuan Zhang, Yan Cong* and Yuanjin Zhao*

**Supporting Figures**


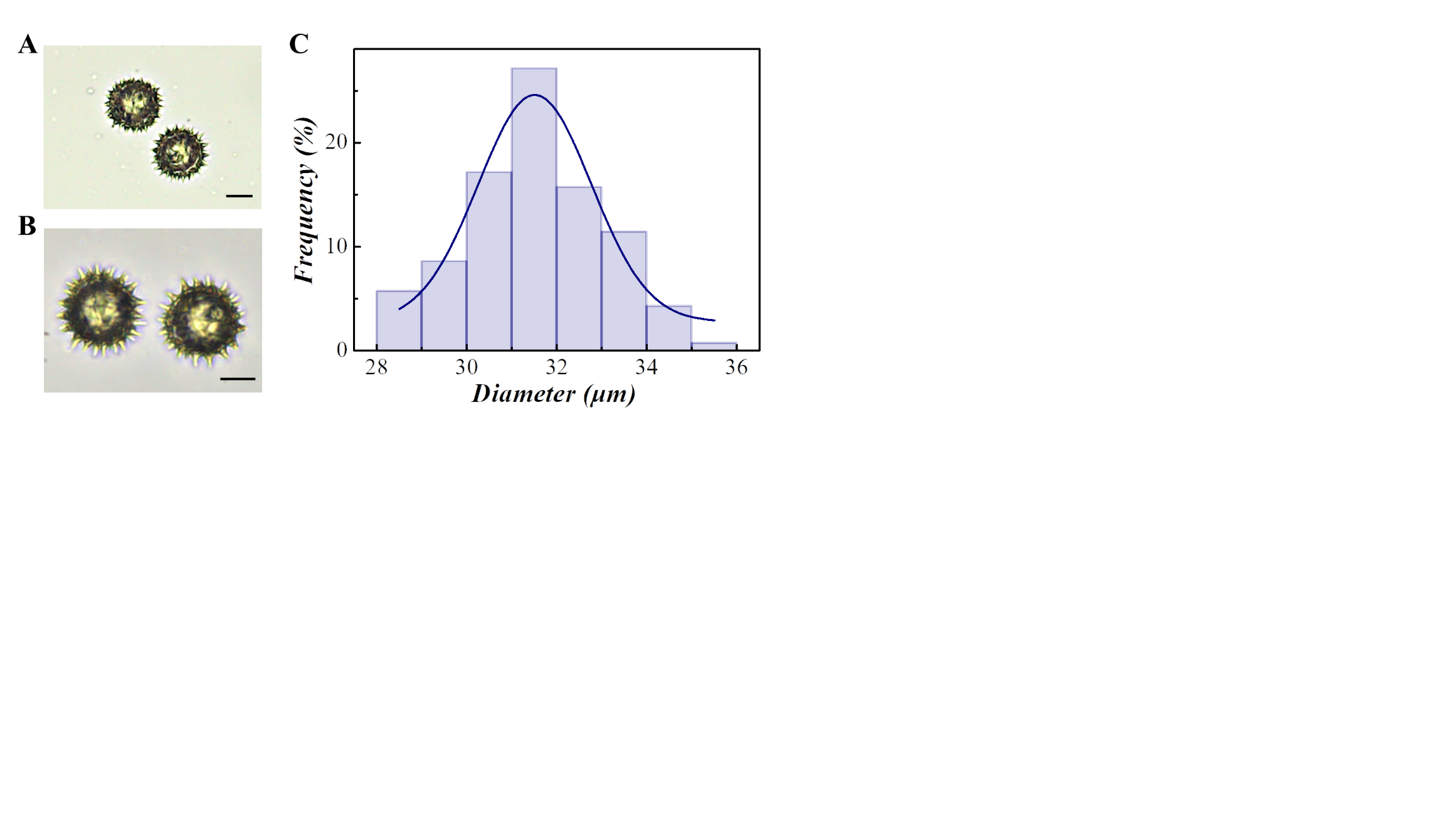


**Figure S1.** (A, B) Optical images of pollen grains. (C) Statistical analysis of TPSs. Scale bars are 20 μm in (A) and (B).


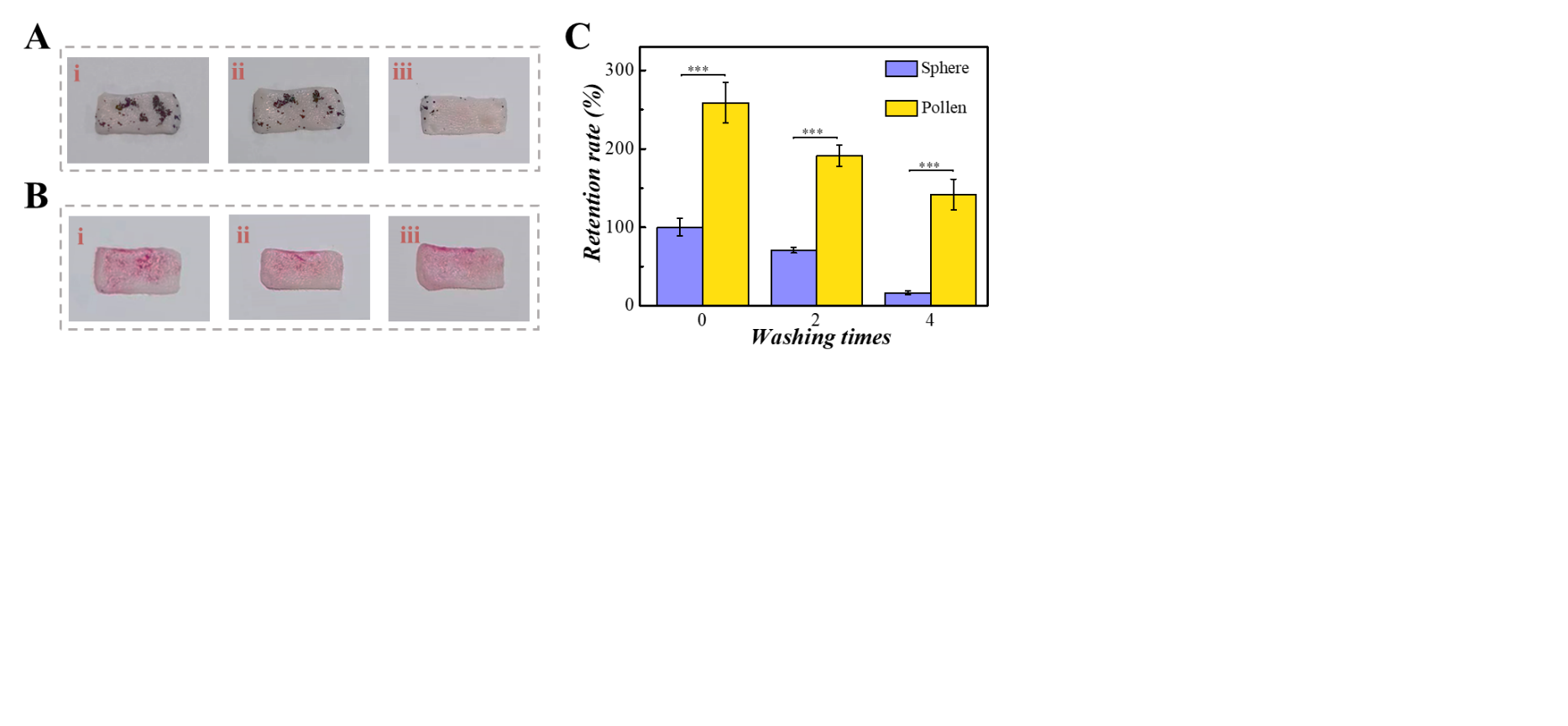


**Figure S2.** Retention test. (A) Images of adhesion conditions on the skin of the spherical microparticles and (B) RhB-loaded TPSs. (C) Statistical analysis of the retention rate. ***P <0.001.


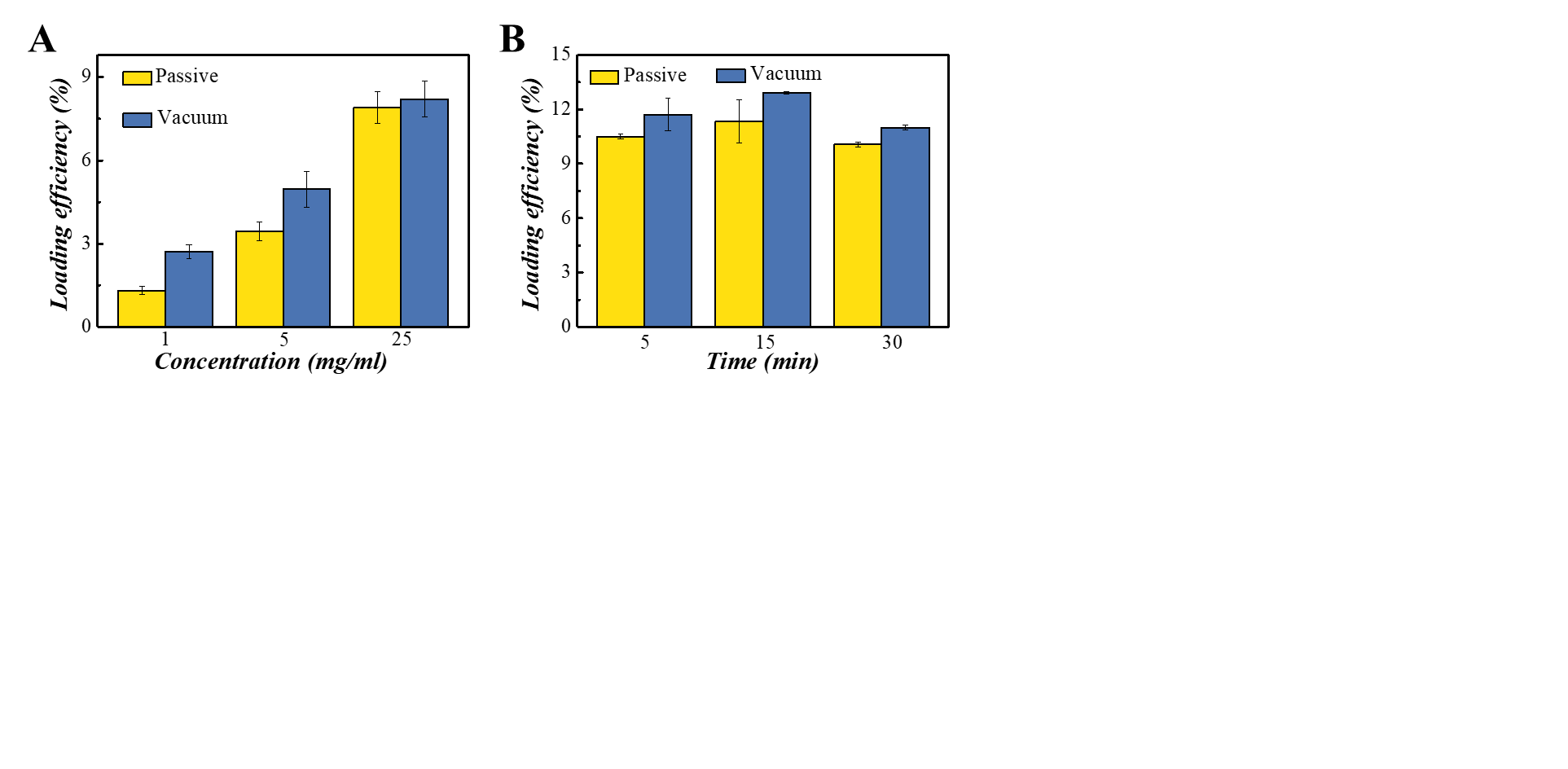


**Figure S3.** Drug loading performance of TPSs. (A) Effect of RhB loading solution concentration on RhB loading efficiency. (B) Effect of time on RhB loading efficiency.


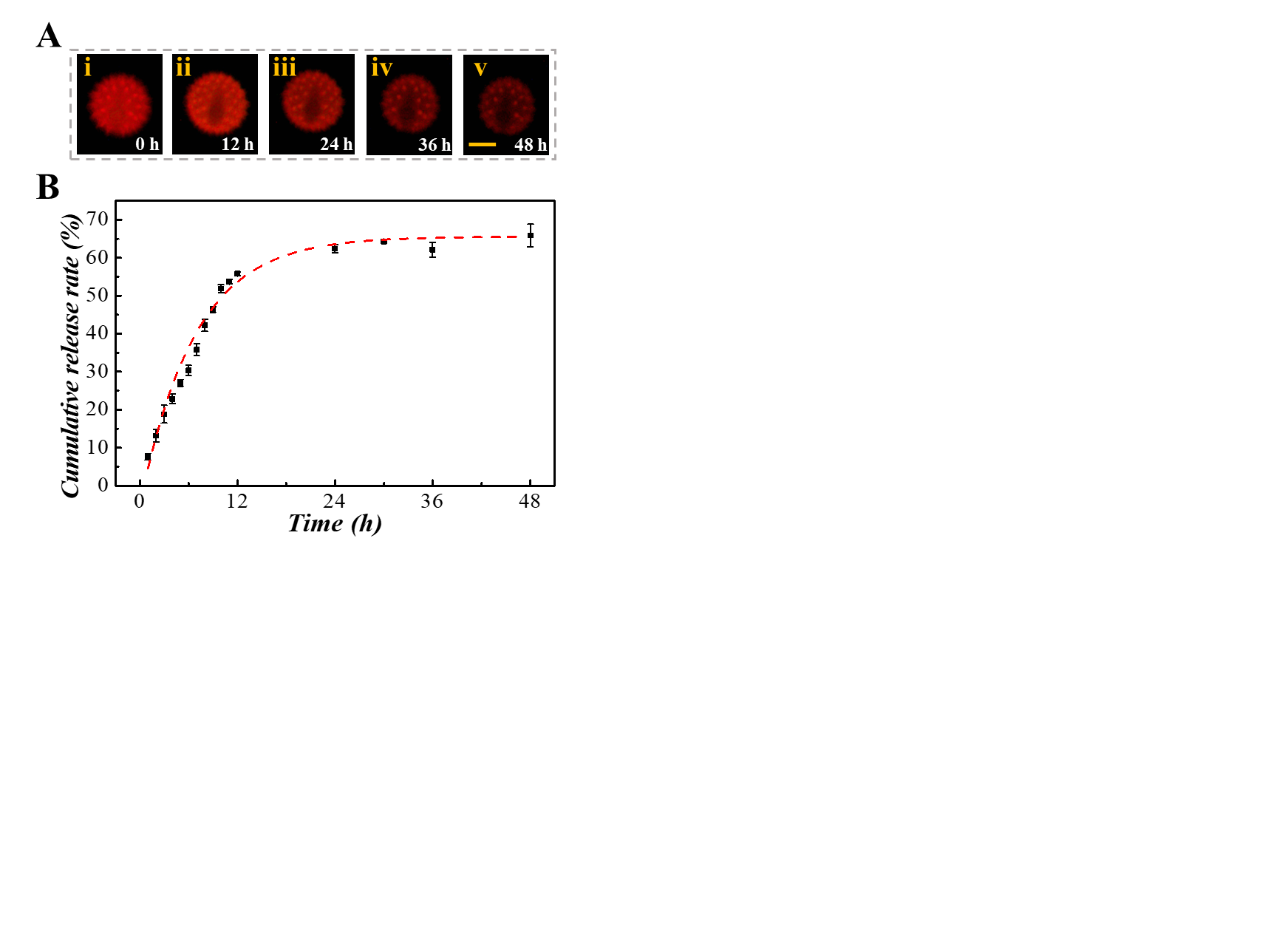


**Figure S4.** Drug delivery performance of TPSs. (A) Fluorescence images of TPSs loaded with RhB during drug release, scale bar is 15 μm. (B) Cumulative release profile of RhB-loaded TPSs.


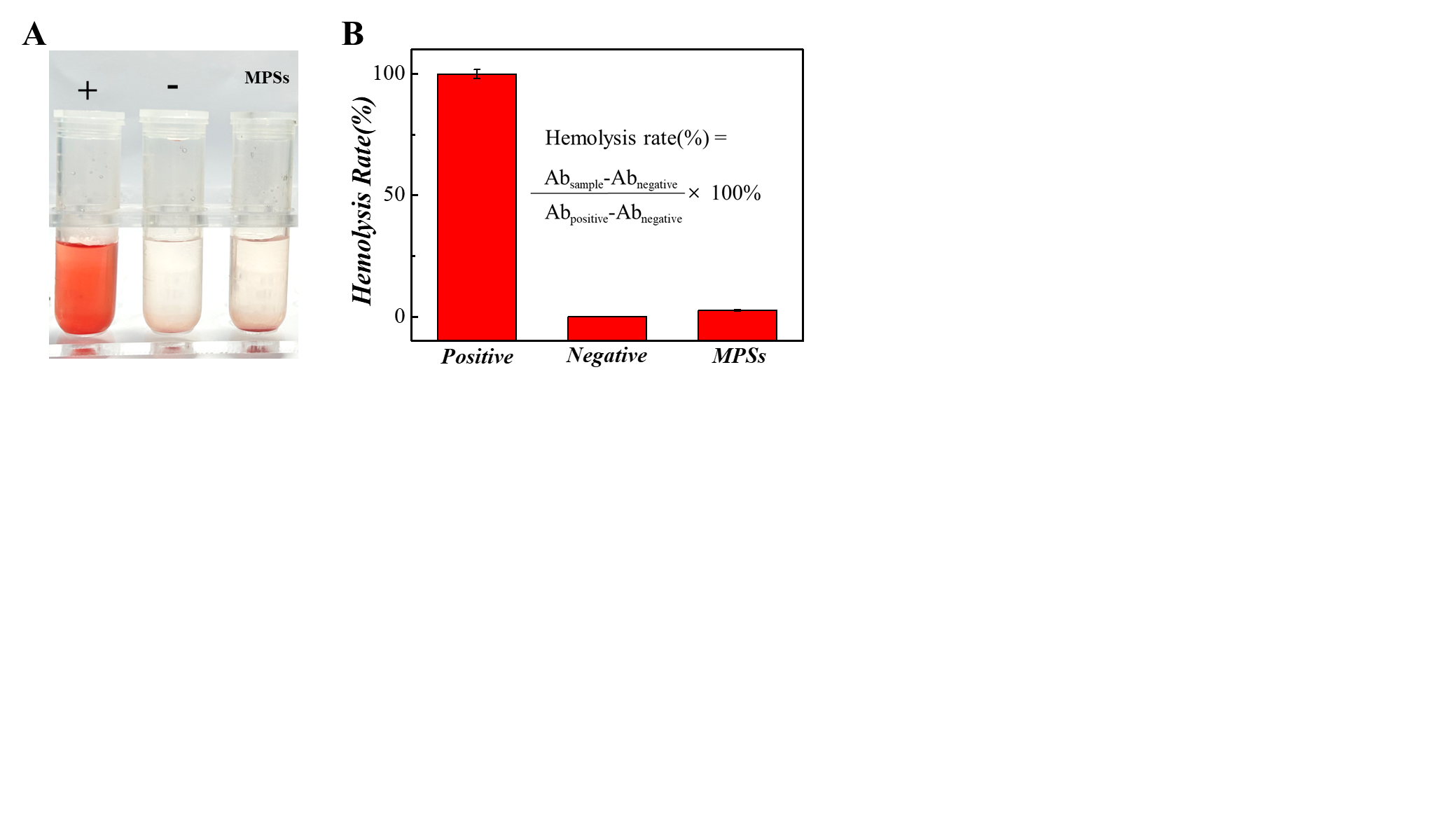


**Figure S5.** Hemolysis test. (A) Image of the result of hemolysis test. (B) Statistic analysis of the hemolysis test.
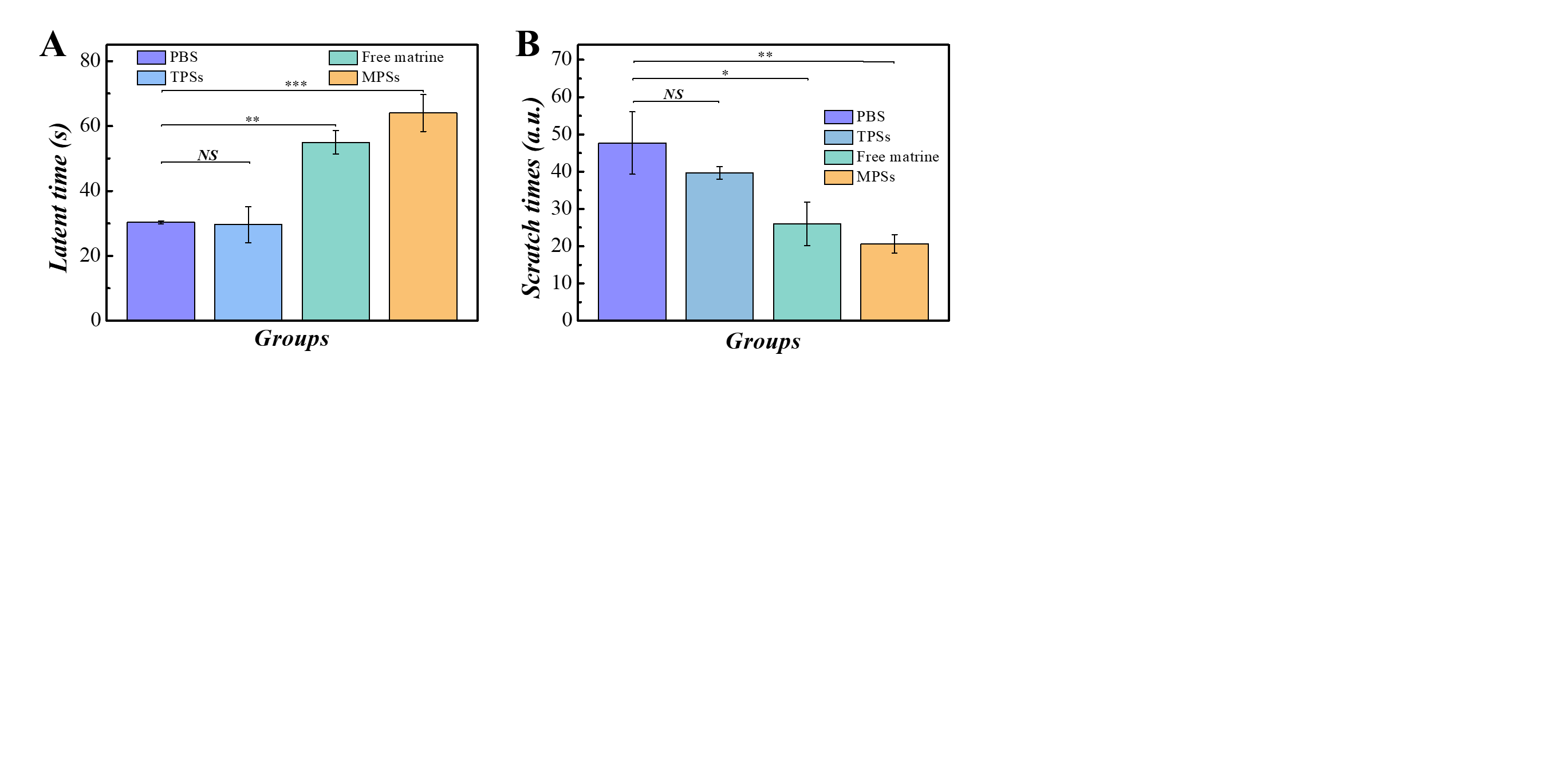


**Figure S6.** Antipruritic test. (A) Statistical analysis of the latent time. (B) Statistical analysis of scratch times. *P<0.05; **P < 0.01; ***P <0.001; NS, not significant.


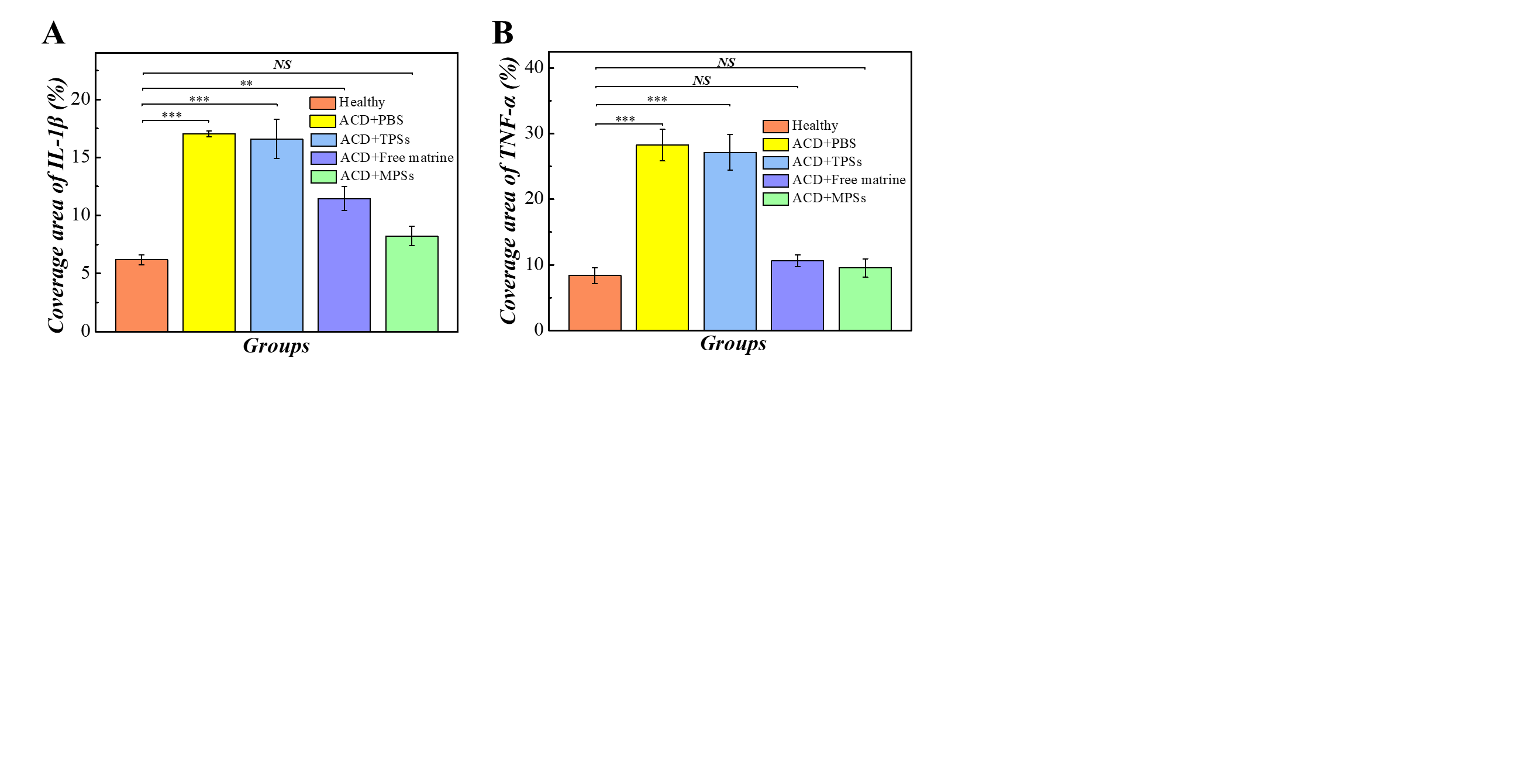


**Figure S7.** Qualitative analysis results of histochemical staining. (A) The statistics of positive coverage area of IL-1β. (B) The statistics of positive coverage area of TNF-α. **P < 0.01; ***P <0.001; NS, not significant.
